# Visual impairment, coping strategies and impact on daily life: a qualitative study among working-age UK ex-service personnel

**DOI:** 10.1186/s12889-015-2455-1

**Published:** 2015-11-12

**Authors:** Sharon A. M. Stevelink, Estelle M. Malcolm, Nicola T. Fear

**Affiliations:** King’s Centre for Military Health Research, King’s College London, Weston Education Centre, 10 Cutcombe Road, SE5 9RJ London, United Kingdom; Academic Department of Military Mental Health, King’s College London, Weston Education Centre, 10 Cutcombe Road, SE5 9RJ London, United Kingdom

**Keywords:** Blindness, Coping, Disability, Health-related quality of life, Military, Well-being

## Abstract

**Background:**

Sustaining a visual impairment may have a substantial impact on various life domains such as work, interpersonal relations, mobility and social and mental well-being. How to adjust to the loss of vision and its consequences might be a challenge for the visually impaired person. The purpose of the current study was to explore how younger male ex-Service personnel cope with becoming visually impaired and how this affects their daily life.

**Methods:**

Semi-structured interviews with 30 visually impaired male ex-Service personnel, all under the age of 55, were conducted. All participants are members of the charity organisation Blind Veterans UK. Interviews were analysed thematically.

**Results:**

Younger ex-Service personnel applied a number of different strategies to overcome their loss of vision and its associated consequences. Coping strategies varied from learning new skills, goal setting, integrating the use of low vision aids in their daily routine, to social withdrawal and substance misuse. Vision loss affected on all aspects of daily life and ex-Service personnel experienced an on-going struggle to accept and adjust to becoming visually impaired.

**Conclusions:**

Health care professionals, family and friends of the person with the visual impairment need to be aware that coping with a visual impairment is a continuous struggle; even after a considerable amount of time has passed, needs for emotional, social, practical and physical support may still be present.

**Electronic supplementary material:**

The online version of this article (doi:10.1186/s12889-015-2455-1) contains supplementary material, which is available to authorized users.

## Background

Becoming visually impaired can be a life changing experience and is likely to have far reaching consequences for the person affected [1–3]. Persons acquiring a visual impairment express a variety of emotional, cognitive, behavioural and social responses to this significant loss. The model of grief proposed by Kübler-Ross, originally used to describe coping in terminally ill persons, has shown to be useful in a variety of settings in which persons face a significant crisis, change or loss, such as acquiring a visual impairment [4, 5]. People affected will mourn their loss of vision and associated losses including their job, leisure activities and independence and may go through various phases of grief, which include denial, anger, depression, bargaining and acceptance [6]. Positive or negative mechanisms of adjustment, also termed coping strategies, can help individuals to master their impairment. Broadly speaking, we can distinguish between adaptive coping strategies such as seeing a counsellor and goal setting or maladaptive coping strategies including social withdrawal and substance misuse [7, 8].

Service personnel are at a higher risk of becoming visually impaired than civilians as a result of deployment experiences. A decrease in combat-related mortality has been reported during the recent deployments to Iraq and Afghanistan compared to other conflicts; this can be explained by technological and medical advances in medical care, body armour and casualty evacuation [9]. However, more Service personnel return home with combat-related trauma such as shrapnel wounds, extremity amputations, head injury and vision loss [9–12].

Recently, a study reviewing the prevalence of mental health problems among (ex-) service personnel with an irreversible impairment (e.g. hearing, vision, but predominantly physical) concluded that common mental health disorders such as post-traumatic stress disorder, anxiety, psychological distress and depression, were frequently reported, but levels varied widely across study populations. Nevertheless these levels appeared to be higher than found in comparable samples of civilian and military populations without an impairment [13].

This study utilised a sample of younger (ex-) Service personnel who are members of Blind Veterans UK (55 years of age or below). Blind Veterans UK is a charity organisation, formerly known as St Dunstan’s, which provides support and care for (ex-) Service personnel who have a visual impairment in both eyes, regardless of the cause. The majority of the members of Blind Veterans UK are 65 years and older, however, due to military operations in Iraq and Afghanistan, the charity has seen an influx in younger members over the last decade. Therefore Blind Veterans UK commissioned the King’s Centre for Military Health Research at King’s College London to examine the mental health and social well-being of their younger members. We explored how ex-Service men adjust to their loss of vision, which coping strategies they use and how their loss of vision and its consequences has impacted on their daily life.

## Methods

### Setting and design

A cross-sectional study was conducted using a mixed methods approach. Phase 1 of the study consisted of telephone interviews with male and female (ex-) Service personnel whereby clinical screening measures for mental health were used. A subsample of the phase 1 participants was invited to join phase 2 of the study. Phase 2 consisted of semi-structured in-depth face to face interviews, covering various topics including the impact of becoming visually impaired on various domains of life and coping strategies. The current paper will report on male participants included in phase 2 of the study.

### Participants

All participants were members of Blind Veterans UK and were under 55 years of age at recruitment. Male participants who took part in phase 1 of this study (*n* = 74) were asked if they would be interested in taking part in a qualitative interview. Sixty-six participants indicated their willingness to do the face-to-face interview. We were especially interested in those who became visually impaired due to their deployment to Iraq or Afghanistan (*n* = 10). They were prioritised in the selection process for phase 2 after which other participants were invited. Thirty male ex-Service personnel were approached and two declined. Therefore, an additional two male members were invited and they agreed to participate. We deemed 30 face-to-face interviews sufficient to reach data saturation based on previous experiences and the literature [14]. The data collection period was closely monitored to ensure that the data collected was rich and descriptive and no new information seemed to emerge whilst approaching the set interview target.

### Materials

A semi-structured interview schedule was used consisting of 11 open-ended questions covering different aspects of how a visual impairment can have an impact on life, difficulties experienced because of being visually impaired and how ex-Service men adjusted to life with a visual impairment (Additional file 1). These questions were developed in collaboration with Blind Veterans UK, to ensure they met the remit of the work commissioned and were appropriate for use in the target population. The draft interview guide was piloted by E.M.M. and S.A.M.S. among two members of Blind who just exceeded the age threshold off 55 years. Feedback from the participants suggested that the questions were received well, easy to follow and comprehensive. Therefore no changes were made to the interview guide. Socio-demographic data obtained during phase 1 were linked to the participants who were included in phase 2 of the study.

### Procedure

Interviews took place between December 2012 and May 2013. Prior to the interview, participants received an information package explaining the study and a detailed signposting booklet (in accessible formats) providing information about various sources of help and advice that might be useful for the participant, such as the Veterans UK helpline.

Participants were interviewed by two researchers (E.M.M. and S.A.M.S.) who were trained and experienced in using the qualitative interview schedule. The majority of the interviews took place in the participant’s home environment after verbal informed consent was given. Procedures were in place for participants who were distressed or at risk and required a call back from a Community Psychiatric Nurse. All interviews were recorded with a Dictaphone. Interviews lasted 35–105 min. At the end of the interview participants were thanked for their time and received £20 in cash.

### Analysis

The interviews were transcribed in full to include all spoken words and non-verbal utterances such as sighs and laughter. Recordings were listened to and transcripts were read and re-read by E.M.M. and S.A.M.S. Both independently coded five different transcripts each using NVivo as an organisational tool. Once five transcripts were independently coded, E.M.M. and S.A.M.S. met to compare and discuss. The initial coding framework was grounded in the content of the data (inductive). Data were analysed thematically [15]. Throughout the first stages of the analysis process the coding framework was revised and further developed. E.M.M. and S.A.M.S. independently read and coded three additional transcripts to ensure the coding framework reflected the data. Any disagreement about codes between the two researchers were discussed and resolved. Once the coding frame was finalised, E.M.M. independently coded all of the transcripts (Additional file 2). Intra-coder agreement was established by E.M.M. by coding three interviews at two time points which were one month apart. An intra-coder agreement of 84.5 % was found, indicating good agreement. Once all transcripts were coded, the codes were put into broader themes and associated sub-themes. The main themes identified for the current paper were coping (strategies) and the impact of vision loss on daily life. Pseudonyms are used when presenting the data.

### Ethical approval

Ethical approval for this study was granted by the Social Care Research Ethics Committee (12-IEC08-0032).

## Results

Out of the 30 younger ex-Service men, 27 were below 45 years of age; 15 were employed at the time of the interview; four were still serving but were waiting to be medically discharged. Of those who had left the Armed Forces, 13 out of 30 had left over 10 years ago and just over half (17 out of 30) were married or in a long term relationship. The great majority (26 out of 30) had served in the Army.

Twelve men had a combat-related visual impairment, of which 10 sustained the impairment during deployment to Iraq or Afghanistan. 12 out of the 30 sustained their impairment less than 5 years ago. Genetic causes of visual impairment were the most common causes of visual impairment (*n* = 7) among those with a non-combat-related visual impairment followed by ocular medical conditions (e.g. age-related macular degeneration, glaucoma) (*n* = 4) and environmental causes (*n* = 4) (e.g. toxic or injury related). The overwhelming majority of ex-Service personnel used low vision aids (28 out of 30); four had a guide dog, 17 used talking books and 19 used a white stick. Approximately one in three participants screened positive for probable depression, probable anxiety or probable Post-Traumatic Stress Disorder (Stevelink et al., (2015) http://bjo.bmj.com/content/early/2015/04/23/bjophthalmol-2014-305986.full.

In the next section the findings of the two themes identified for the current paper, specifically ‘coping (strategies)’ and ‘impact of vision loss on daily life’, are combined to describe how vision loss affected the person from the time directly after becoming visually impaired and how this changed subsequently.

### Coping with a visual impairment and impact on daily life

Directly after becoming visually impaired, younger male ex-Service personnel thought *“life is over”* and they “*[didn’t] want to carry on”*. Their confidence was undermined, they felt sorry for themselves and had the feeling there was no way out.

Phil (non-combat-related visual impairment, age 35–44 years)*: “Well initially straightaway it* [loss of vision] *stopped me from going out straightaway. I went in for the first two years, first year and a half at least, I was very depressed. Very sorry for myself and thought that was it (…). (…) I didn’t think there was anything I could do so yeah, dread, full of dread and fear and all that lot did come into it.”*

These feelings and experiences were reinforced by other losses that were experienced as a result of their loss of vision such as losing their job, experiencing relationship difficulties and an increased dependence on others.

In most cases, if personnel suffered a deterioration of their sight whilst in Service, they were medically discharged and as a result had to confront the issue of changing their career. This was similar amongst those who had left the Service and had started a civilian job. This forced change of career was generally experienced as a *“regression”* leading into a cascade of accompanying consequences such as experiencing financial hardship if living on benefits, different family dynamics due to for example family members taking on the role of carer and, above all, impaired self-esteem. Ex-Service men felt that by no longer being the breadwinner in the household and a highly trained professional, they were set back to square one as said by Alan (combat-related visual impairment, age 25–34 years):

*“(…) it’s like all that experience, all that knowledge… shoved right back in your face (…). (…) You’re a broken toy now. What happens to broken toys… goes to the tip doesn’t it? (…). You know you’re a broken toy they don’t want to know you.”*

Denial of the consequences of vision loss resulted in ex-Service men trying to do the things they were used to do; this resulted in feelings of frustration and irritation as illustrated by Richard (non-combat-related visual impairment, age 45–54 years):

*“(…) at first it* [loss of vision] *made me really down and depressed and … . Because I’d known for quite a while before that there was something wrong. And I went through not accepting it. So at first I wouldn’t accept it and then when I first finished work obviously I had to walk everywhere. I got quite narky with people. If I was out with my stick and they’d bump into me. I’d get quite angry with them… because it was everybody else’s fault for getting in my way. So I went through like a stage of denial, I suppose and then being angry.”*

The emotional turmoil personnel went through whilst adjusting, adversely affected their relationship with their partner. Those who were in a relationship at the time of becoming visually impaired suggested that their impairment put a strain on that relationship. Personnel experienced increased dependence on their partner resulting in changing relationship dynamics. Both the partner and the person affected needed time to adapt to this new situation. In a few cases, participants divorced or ended their relationship with their partner, but the dominant view was that the impairment was a contributing factor but not the main reason for breaking up.

Charlie (non-combat-related visual impairment, age 35–44 years): “*But it* [loss of vision] *very nearly I think cost us my wife and I our marriage, because I was quite unpleasant on more than one occasion. But thankfully we’re coming through the other side so yeah it’s been a difficult journey.”*

Members who became visually impaired in combat were proud about the circumstances that led to their loss of vision (*“serving Queen and Country”*), whereas those with a non-combat-related visual impairment struggled with the question ‘why me?’ and even felt guilty, ashamed or embarrassed. Some personnel expressed the hope that their vision would improve over time or were looking into potential treatment options. Personnel were mourning about what they had lost and what they could have done if they had not sustained a visual impairment.

Harry (combat-related visual impairment, 35–44 years):* “You know the doctors are going to say to you at some point you don’t need to come and see us anymore, and that’s when it will sink in and that’s what you’ve got for the rest of your life, that’s what you’re stuck with. And at that point you need to just accept it, just get on with it because the longer you kid yourself it’s going to get better, or there’s going to be some miracle surgery, the longer it ‘ll take you to adapt.”*

The visual impairment not only affected the domain of work and interpersonal relationships but also other areas as illustrated by Nick (non-combat related, age 25–34 years):

“*(…) you do have a bad impact on your day to day life especially from washing up to having food or to prepare a meal. Then taking medication and also you know dress yourself, and also you can’t actually go out on your own all the time. So your movement is quite restricted although it can be done with some training outside, but there is the danger off (…) colliding with something or someone or some obstruction.”*

As time passed, ex-Service personnel were able to “*change [their] head around*” and tried to adjust to their visual impairment and its consequences, by applying various coping strategies. The ‘military ethos’ of *“crack on”* and *“adapt and overcome the situation”* helped them to overcome any problems they experienced. However, for some personnel it acted as a barrier because they were reluctant to ask for help and struggled through with their visual impairment (defined by the researchers as coping at a cost).

Other reasons for coping at a cost were that younger ex-Service personnel felt ashamed, lacked confidence or were too proud to ask for help. They did not want to be seen as a burden on others. Coping at a cost was enforced by reactions from the public. For example, Max (non-combat-related visual impairment, 35–44 years) was assaulted when using his cane on public transport after accidently bumping into someone; they did not believe he was visually impaired so from that moment on he decided not to use a cane anymore.

The unavailability of support and resources influenced how younger members coped with their loss. Ex-Service personnel tried ‘to escape’ by, for example, substance abuse or made a non-fatal suicide attempt.

Tom (non-combat-related visual impairment, 25–34 years): “*When I first lost my eyesight I never had that emotional support. I never had it and I dealt with it on my own. My way was hitting the drinks and hitting the drugs and going crazy*.”

Other examples of maladaptive behaviour included isolation and social withdrawal from family and friends, acting aggressively or in an unfriendly manner.

Besides the use of maladaptive coping strategies, several adaptive coping strategies were applied. One of these was termed by the researchers as ‘downward comparison’. Younger ex-Service personnel pointed out that they were aware of other people being worse off than themselves such as soldiers with serious brain injury, cancer patients or if they still had some vision left those who were completely blind. Others made a comparison with vision impaired members who managed to carry on successfully. By making a downward comparison or by comparing themselves with people who had faced the same problems, coping was facilitated as members got inspired and motivated to *“crack on with life”.*

Oliver (non-combat-related visual impairment, 35–44 years): *“They* [members of Blind Veterans UK] *proved that there are other people in the same situation as me and even not worse, and that you know you can still do day to day tasks and you can still do a lot of varied things if you put your mind to it. You know and it’s just challenging your mind to being able to do these things.”*

People’s favourite leisure activities and interests changed substantially because they were no longer able to undertake them due to their loss of vision. Driving a car was missed tremendously, followed by sporting activities and reading. Personnel tried to get around these barriers by adopting a problem-focused approach (e.g. use of low vision aids, retrain, find other activities they were able to do). Also accepting or asking for (social) support from family, friends, charities or seeking professional support, helped ex-Service personnel to adjust and carry on.

Just after becoming visually impaired, personnel needed a lot of help and relied heavily on others. Once they started to adapt, people learned new skills and strategies, resulting in increased confidence and less reliance on others. This had a positive impact on the mental well-being of the person with the visual impairment and facilitated the process of accepting their loss of vision and its consequences. Further personnel learned what they are still able to do and what not, thereby reflecting on and adapting to the restrictions their visual impairment imposed. However, the dependence on others played a limiting role and new activities such as taking up different hobbies like disability sports were not always experienced as that satisfying.

Andrew (combat-related visual impairment, 25–34 years): *“You have to accept that sort of thing as part of the hard bit in the beginning. (…) once you’ve accepted it* [loss of vision] *you get used to it and just move on from then. Then obviously you have achievement from there and then you put yourself to whatever you need to achieve.”*

Achievement was often mentioned as a next step. Younger ex-Service personnel decided to set a particular goal such as starting a new course or degree, or signed up for a sporting challenge. By working towards a particular goal, personnel got back in a daily routine, their confidence increased as well as their self-worth. This impacted positively on their mental well-being. Reflecting on the different experiences of the participants it becomes apparent that coping with loss of vision is a dynamic long-term process. Even after years, people may struggle as new situations, challenges or life events come by.

Jack (combat-related visual impairment, 25–34 years): *“It’s been* [amount of years] *now and there was a time I thought I’d accepted what had happened to me, but you know that was temporary. And yeah I don’t think I’ve fully accepted what’s happened to me. There [are] good periods and bad periods and you know they come and go quite randomly and yeah they affect me for different amounts of time and I can get quite negative sometimes and quite self-deprecating.”*

## Discussion

Younger ex-Service men suggested that becoming visually impaired had turned their life upside down; the accompanying consequences had adverse effects on a variety of life domains and adjusting was experienced as a tough journey. Personnel struggled with an increased level of dependence on others, a loss of freedom, a lack of confidence and impaired feelings of self-worth. Various coping strategies were applied by younger ex-Service men and these enabled them to adjust to their loss and overcome challenges they faced down the line. Coping was an on-going and dynamic process with ex-Service personnel experiencing good and bad times, even after many years since sustaining their impairment.

### Coping

The Kübler-Ross model was initially developed to describe how people cope with death and dying following a distinct 5 stage model (denial, anger, depression, bargaining and acceptance) [6]. We used this model as an example in the introduction to describe how people may deal with facing a significant loss, such as sustaining a visual impairment. However, over the years this model has been critiqued widely [16–18].

When reflecting on our results, it became clear that ex-Service men experienced a far wider range of emotions and behaviours; in some cases these emotions occurred simultaneously. Instead of a stage like pattern of response, the data showed coping was more dynamic, highly interactive and a unique journey for every individual. Whereas the individual affected plays a central role in Kübler-Ross’ model, we found that other factors played an important role as well as to how people adjust, including the availability of social support, use of low vision aids, presence of public attitudes, family composition and other situational factors [19]. The variety of positive or negative mechanisms of adjustment helped the individual to master their impairment and should be referred to as forms of coping strategies [18]. Acceptance is described as the closing stage of the grief process by Kübler-Ross. However, as pointed out by Murray and colleagues (2010), and reflected in our data, we found coping to be a long-term process, whereby the person affected will apply different strategies depending on the changes or new situations they encounter [20]. Therefore, acceptance may occur temporarily but will interact with new periods of adjustment and the grief process can be characterised as ‘a chronic recurrent but episodic process’ [20]. This has been illustrated earlier with a quote from Jack (combat-related visual impairment, 25–34 years): he interchangeably experiences periods of acceptance vs. rejection, despite the fact that a considerable amount of time has passed by since he became visually impaired.

Personnel tried to adjust using various strategies, including a strategy termed by the researchers as ‘coping at a cost’. Underlying reasons such as feeling too embarrassed to ask for help, not wanting to burden others or wanting to ‘crack on’ alone. Participants suggested that asking for help would impede on their sense of self-pride and self-respect [19, 21–24]. This attitude of ‘cracking on’ is a key element of the work ethic of the British Army and encourages pro-activity over passivity and contemplation [25]. A different strategy was comparing themselves with others they classified as worse off [26, 27]. This downward comparison motivated and inspired people to life their live to the fullest. For ex-Service personnel with a combat-related visually impairment, feelings of being grateful to still be around were apparent. Analysis of the quantitative data of the current study suggested that the prevalence of mental health problems was not substantially different between personnel with a combat-related visual impairment (25.0 %) compared to those with a non-combat-related visual impairment (29.6 %) (Ref).

### Impact of visual impairment on life domains

Various quantitative studies have examined the impact of vision loss on the psychological well-being of elderly populations [3]. A review summarizing qualitative evidence of seventeen studies on the impact of and coping with vision loss suggested that people relied more heavily on others to perform their daily activities [7]. Further, they reported the loss of leisure activities and hobbies. In addition, a negative impact on mental well-being was described such as the onset of depression, impaired self-esteem, being less socially active and experiencing challenges with regards to interpersonal relationships and communication [7]. Despite the review summarizing studies among the elderly, the findings correspond well with those from the current study. However, the researchers would like to highlight that while the actual barriers and changes due to the loss of vision and its related consequences may be unique for each person, they impede on the same highly valued core concepts of (in) dependence, autonomy, self-esteem, control, confidence, freedom and identity.

A different review synthesised the findings of quantitative studies about the psychosocial impact of vison loss in adults aged 18–59 years [2]. They concluded that the general mental health, social functioning and quality of life were adversely affected. However, this was not consistent for the impact of visual impairment on the prevalence of depression [2]. The association between depression and visual impairment appears to be more evident among elderly [1, 24].

### Limitations

This study had various limitations. First, all participants were members of the charity organisation Blind Veterans UK and had served in the UK Armed Forces. In what way these findings can be extrapolated to different visually impaired populations such as civilians, elderly, women and people who are not involved in a charity organisation remains uncertain. Second, the research team asked sensitive questions related to how personnel coped with their visual impairment. They could have given socially acceptable answers because they did not feel confident, did not trust the interviewer or did not want to appear as ‘weak’. This issue was addressed by informing them what to expect, building good rapport and providing the opportunity to ask the team questions. An important strength of this qualitative study is the substantial sample size (*n* = 30). Therefore, we are confident that we captured a wide range of views and experiences.

### Implications

Clinicians, ophthalmologists and other health care providers as well as partners, the wider family and friends need to be aware that coping is an on-going process and that even after a considerable amount of time, needs for emotional, social, practical and physical support may still be present. Functional training and psychosocial support should be offered enabling personnel to regain their confidence, feelings of self-worth and independence.

## Conclusions

Our findings suggest that sustaining a visual impairment is a life changing event that has important effects on various domains of life. Adjusting to loss of vision and its related consequences can be described as a long-term and difficult journey whereby ex-Service personnel apply various coping strategies to overcome the challenges they face later on in their lives. Future research should be directed into how positive coping can be facilitated and how factors such as the duration of the visual impairment and perceived social support, influence coping processes.

## Additional files

Additional file 1:
**Coding framework with the two main themes identified for the current paper.** (DOCX 15 kb) Additional file 2:
**Semi-structured interview guideline.** (DOCX 14 kb)
