# Supplementary material for: Visual impairment, coping strategies and impact on daily life: a qualitative study among working-age UK ex-service personnel
Source: BMC Public Health. 2015 Nov 12;15:1118. doi: 10.1186/s12889-015-2455-1 (PMC4643496; doi:10.1186/s12889-015-2455-1)
Supplement: Additional file 1: — Coding framework with the two main themes identified for the current paper. (DOCX 15 kb) [file 12889_2015_2455_MOESM1_ESM.docx]

**Additional file 1**

**Coding framework with the two main themes identified for the current paper**

| **Code** | **Description** |
| --- | --- |
| *Coping (strategies)* |  |
| 5 stage model of grief | Feelings and behaviours people may experience whilst adjusting to their loss of vision: denial, anger, bargaining, depression and acceptance. |
| Military attitude | Common attitudes, perceptions and behaviours reflecting the military culture |
| Coping at a cost | Displayed behaviours, perceptions and attitudes that impeded people from asking help if needed |
| Downward comparison | Displayed behaviours, perceptions and attitudes whereby people compared themselves with others who were in their eyes worse off |
| Response shift theory | Displayed behaviours, attitudes and perceptions reflecting the process that after the catalyst (loss of vision) and its impact on daily life, adjustment occurs using mechanisms that bring about a new perceived quality of life |
| Humour, staying positive | Displayed behaviours, perceptions and actions that show positivity and optimism in dealing with the situation |

| **Code** | **Description** |
| --- | --- |
| *Impact of vision loss on daily life* |  |
| Impact on mental wellbeing | Describes how loss of vision impacts on the mental wellbeing of those affected |
| Impact on daily living, household chores and mobility | Describes how loss of vision impacts on daily living activities such as doing household chores, self-care, getting around in the house and in the community |
| Impact on work, finance and studies | Describes how loss of vision impacts on work, career, financial situation |
| Impact on family | Describes how loss of vision impacts on other family members such as children, but also on family dynamics and role expectations |
| Impact on relationships / marriage | Describes how loss of vision impacts on intimate relationships, their relationship with their partner/spouse |
| Impact on sense of identity | Describes how loss of vision impacts on how people perceive themselves, their expectations about what they should (be able) do, their ability to fulfil particular roles (being a dad, partner etc.) |
| Impact on social life | Describes how loss of vision impacts on social and group activities like going out with friends, doing leisure activities, playing sports, socializing |
