# Supplementary material for: Visual impairment, coping strategies and impact on daily life: a qualitative study among working-age UK ex-service personnel
Source: BMC Public Health. 2015 Nov 12;15:1118. doi: 10.1186/s12889-015-2455-1 (PMC4643496; doi:10.1186/s12889-015-2455-1)
Supplement: Additional file 2: — Semi-structured interview guideline. (DOCX 14 kb) [file 12889_2015_2455_MOESM2_ESM.docx]

**Additional file 2**

**Semi-structured interview guideline**

1. Tell me about what impact your visual impairment has had on your life.

(Prompt: to include mental and social wellbeing, family life/marriage/children, sense of identity)

1. What's the hardest thing about being visually impaired for you?
2. What or who has helped you to get through this?

(Prompt: What support have you received from Blind Veterans UK, from other charities, form other support groups? What type of services have you tried or used to assist you with dealing with your visual impairment?)

1. What has been helpful?

(Prompt: What's been the most helpful intervention you've received?)

1. What could have been better?
2. What support would you like that you currently don't have?
3. How have your needs changed over time?
4. What are you main worries about the future with regards to your VI?

(Prompt: how do you think your needs will change in the future?)

1. What's the one thing that no-one asks about/knows about in terms of life as a VI person?
2. Have there been any positive changes which have come about as a result of this loss?
3. If you met a veteran who had recently become visually impaired, what's the best advice you could give them?
